# Risk factors for measles outbreak in Ataq and Habban districts, Shabwah governorate, Yemen, February to May 2018

**DOI:** 10.1186/s12879-021-06207-3

**Published:** 2021-06-10

**Authors:** Abdulkareem Ali Hussein Nassar, Mohammed Abdullah Al Amad, Mohammed Qasim, Fekri Dureab

**Affiliations:** 1 Sana’a City’s Public Health and Population Office, Yemen Ministry of Public Health and Population, Sana’a, Yemen; 2 Field Epidemiology Training Program, Yemen Ministry of Public Health and Population, Sana’a, Yemen; 3 Evaluation and Monitor Department, World Health Organization- Yemen, Sana’a, Yemen; 4 Heidelberg Institute of Global Health, Medical School , Heidelberg University, 69120 Heidelberg, Germany; 5 IRIA, Akkon-Hochschule fuer Humanwissenschaften, 12099 Berlin, Germany

**Keywords:** Measles outbreak, Risk factors, Shabwah governorate, Yemen

## Abstract

**Background:**

Recent conflict and war in Yemen lead to collapse of the health system, decrease of immunization coverage and spread of many outbreaks. On May 22, 2018, the surveillance officer in Shabwah governorate reported an increased number of suspected measles. On May 24, 2018, a team from Yemen-Field Epidemiology Training Program was sent to investigate. The aims were to describe the outbreak, determine the risk factors for measles infection and recommend control measures.

**Methodology:**

A descriptive followed by case-control study design (1:2 ratio) were performed. National Measles Surveillance Program case definition and predesigned questionnaire were used to collect data from 73 cases and 146 controls. Attack rate (AR), adjusted odds ratios (aOR) and 95% confidence intervals (95%CI) were calculated. *P* value < 0.05 was considered as the cut point for significant. Epi info version 7.2 was used.

**Results:**

A total of 73 suspected cases were found. Almost 53% were from Habban district, 63% were males and 56% were among age group < 5 years. The overall AR was 82/100,000 population. Measles was significantly associated with contact with case (aOR = 27.3, 95% CI:1.3–551.7), malnourished children aged 6–60 months (aOR = 24.9, 95% CI;1.9–329.6) and unvaccinated children (aOR = 17.2, 95% CI:2.9–100.7). The six collected blood samples found to be positive for measles IgM.

**Conclusions:**

Measles outbreak in Ataq and Habban districts was confirmed. Contact with measles cases, malnutrition and un-vaccination were the potential contributing factors of measles outbreak in Shabwah governorate. An urgent vaccination campaign with health education interventions are highly recommended. Reactivation of the outreach immunization services and strengthening surveillance and response systems are top priority to take place at district and governorate levels.

## Background

Measles is a highly infectious human diseases caused by the measles virus that belongs to the family Paramyxoviridae and frequently results in widespread outbreaks [1, 2]. It is often characterized by high morbidity and mortality rates globally, particularly in African and other developing countries. Mortality from measles infection is often secondary to severe complications [3]. Complications of measles include severe pneumonia, diarrhea, blindness, deafness and encephalitis [1, 3].

Globally; in 1980, before widespread vaccination, measles caused more than two million deaths each year [4]. As a result of the global effort to eradicate the measles virus by vaccination, approximately 20 million measles deaths have been saved. Using an effective vaccine reduced the deaths number from 550,000 to 90,000 during 2000 to 2016 [1, 5], the majority from developing countries, which accounted for 5% of all under-five mortality [1]. However, measles is still one of leading causes of death among children globally [4].

Despite the number of measles cases reduced in The East Mediterranean Region (EMR) in the latest years, it’s still a problem in the region. The number of reported suspected measles were 34,835 in 2015 and 28,225 in 2016 while the laboratory confirmed measles were 8853 in 2015 and 5201 cases in 2016. In 2016, measles incidence per 1000,000 populations was higher in Sudan (42.94), followed by Oman (34.66) and United Arab Emirates (23.92) [6].

Collapse of the health system due to the ongoing war in Yemen lead to low vaccination coverage [7], that reported by 64% during 2017 in all Yemeni governorates [8]. The reported measles cases from January to May 2018, a total of 4474 measles and 91 deaths were reported compared with 3529 measles and 8 deaths were reported through 2017 [9, 10]. Many outbreaks were investigated in some governorates. Such as Hajjah governorate in 2012 [11] and Al Hudaydah governorate in 2014 [12], Al Jawf and Sa’adah governorates in March 2015 [13, 14], and Amran governorate in 2017 [15]. The measles outbreaks still have occurred in 2018. Yemen remains under threat of measles epidemic due to collapse of the health system and continuing of the war.

On May 22, 2018, Shabwah governorate surveillance officer notified the Ministry of Health about an increased number of measles cases in the governorate. On May 24, 2018, Yemen Field Epidemiology Training Program sent a team to perform an investigation. Moreover, studying the risk factors will provide a valuable information to be used for better understanding and control the measles outbreak. The study aims to confirm the existence of measles outbreak in Ataq and Habban districts, describe the characteristics of measles outbreak by person, place and time, determine the risk factors of measles outbreak, and recommend the appropriate control and prevention measures.

## Methodology

### Study design and area

A descriptive followed by case-control study design was conducted in Ataq and Habban districts, Shabwah governorate, Yemen. Shabwah is located in the southeastern part of Yemen, along the Arabian Sea coast. It is 474 km southeast of the capital city of Sana’a. It has 632,000 populations distributed in 17 districts [16].

Based on electronic Integrated Disease Early Warning System data, Ataq and Habban districts were the most affected districts in the governorate [9, 17]. Descriptive part of this study was conducted to describe the characteristics of measles outbreak by person, place and time, while a case–control part was conducted to identify the risk factors associated with measles outbreak.

### Sample size

A sample size of 219 with ratio 1:2 (73 cases and 146 control) were included from Ataq and Habban districts. As a result of security reasons, and the limited resources and period for investigation, an obtainable and convenience sample size was determined as 55% of 132 reported measles cases in Ataq and Habban districts.

### Selection of cases and controls

The National Measles Surveillance Program case definition (any person who lived in Ataq or Habban district, suffered from fever and skin rash since February 2018) was used to identify the cases.

Control defined as: any person who lived in Ataq or Habban district, did not suffer from fever nor skin rash since February 2018. The sex of controls units was matched with the cases.

### Data and laboratory sample collection

Active search from house to house was performed. A predesigned questionnaire was adopted from literatures [18–22]. The questionnaire was used to collect data related to the following variables: sociodemographic, onset date, clinical symptoms, vaccination status (based on vaccination cards or parents recall), contact with suspected case and presence of suspected case in the same area. Also it included questions related to parents’ education level, nutritional status for children aged 6–60 months (mid-upper-arm circumference) [23], house ventilation, distance between home and health facility, and number of family members and rooms. Six blood samples were collected and sent to the National Center for Public Health laboratory to be tested by ELISA.

### Data processing and analysis

Data was cleaned and entered into Excel program and then analyzed by Epi info version 7.2. The distance between home and health facility, and crowding (number of persons per room) were categorized by mean. Nutritional status of children aged 6–60 months were categorized according to the cut-off point of mid-upper arm circumference at 115 mm: < 115 mm as malnutrition and ≥ 115 mm as normal [23]. Family size categorized by average household size in Yemen into < 7 or ≥ 7 persons [24]. Vaccination status were categorized into un-vaccinated (those who have never received a vaccine dose) and vaccinated (those who have received at least one dose of measles vaccine). The Attack Rate (AR) was calculated as the total number of measles cases divided by the total population and expressed per 100,000. Univariate and multivariate binary logistic regression were used to calculate the crude Odds Ratio (cOR) and the adjusted Odds Ratio (aOR) with 95% Confidence Interval (CI). Each variable that has *P* value ≤0.25 was entered into multivariate analysis. P value < 0.05 was considered as the cut point for statistically significant in multivariate analysis.

## Results

**Fig. 1:**
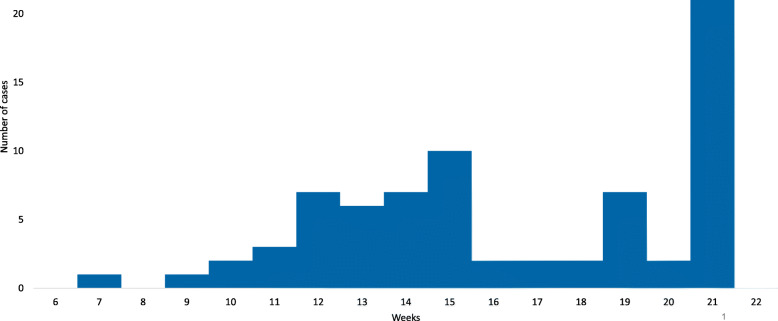
Distribution of measles cases by week of onset, Shabwah governorate, February–May 2018

A total of 73 measles cases were found in both Ataq and Habban districts. The first case was in Epidemiological week number 7. There were two peaks during the course period of the outbreak; the first peak was in epidemiological week number 15 and the second peak was the highest peak occurred in epidemiological week 21 (Fig. 1).

Table 1 shows that 56% of cases were among the children under-5 years old and 62% were males. About 53% of cases were from Habban district and 47% from Ataq district. The overall AR was 82/ 100,000 of population. The AR was higher among the children under- 5 years old (288 vs 43 / 100,000). Similarly, the AR was higher among males than females (94 vs 68/100,000) and in Habban than Ataq district (98 vs 69/100,000).

**Table 1:** Age, sex and district specific attack rates of measles cases, Shabwah governorate, February–May 2018

Variables	Population	Cases No. (%)	Attack rate (per 100,000)
Age group (years)			
< 5	14,247	41 (56)	288
≥ 5	75,034	32 (44)	43
Sex			
Male	47,859	45 (62)	94
Female	41,422	28 (38)	68
Districts			
Habban	39,709	39 (53)	98
Ataq	49,572	34 (47)	69
Total	89,281	73 (100)	82

**Fig. 2:**
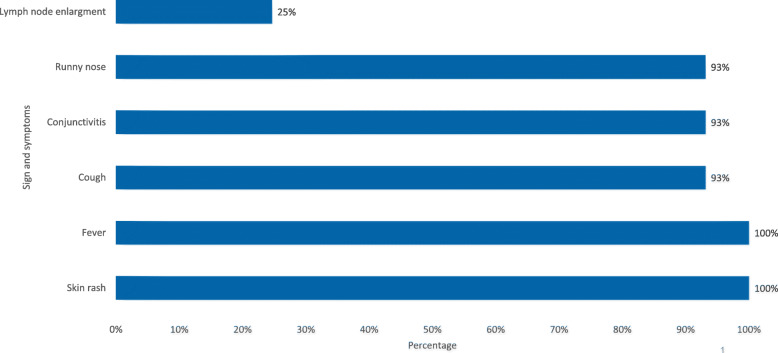
Distribution of measles cases by signs and symptoms, Shabwah governorate, February-May 2018

Figure 2 shows that all cases suffered from fever and skin rash, and 93% were suffered from cough, conjunctivitis and runny nose. However only 25% of the patients had lymph node enlargement. Only six blood samples were collected randomly from the patients and all were positive for measles IgM.

Table 2 shows the distribution of potential risk factors for measles by study group, the children under-5 years old, illiterate or basic education level of parents, malnourished children, unvaccinated children, and previous contact with measles cases were significantly associated with being a case. The findings show the non-adjusted and adjusted findings from the logistic regression analysis of factors associated with measles cases. In the univariate analysis, the children under-5 years old were 2 times more likely to be cases (cOR = 2.4, 95% CI:1.3–4.2), while those who had parents with basic education or totally illiterate were more likely to be a case of measles among fathers (cOR = 2.2, 95% CI:1.2–3.9) less likely than mothers (cOR = 4.7, 95% CI:1.1–20.8). Moreover, cases were found more likely among malnourished children aged 6–60 months (cOR = 4.8, 95% CI;1.2–18.7), unvaccinated children (cOR = 19.9, 95% CI: 9.3–43.1), contacted with measles case (cOR = 37.3, 95% CI: 14.0–99.2) and the presence of measles case in the same area (cOR = 17.9, 95% CI: 8.5–37.7). As well as bad ventilated houses (cOR = 5.5, 95% CI:2.9–10.5) and living in crowded rooms (cOR = 1.8, 95% CI: 1.0–3.2).

**Table 2:** Factors associated with measles outbreak, Shabwah governorate, February–May 2018

Variables	Case No. (%) (*n* = 73)	Control No. (%) (*n* = 146)	Crude OR (95% CI)	Adjusted OR (95% CI)
Age group				
< 5 years	41 (56)	51 (35)	2.4 (1.3–4.2)	1.4 (0.2–8.1)
≥ 5 years	32 (44)	95 (65)		
Father education				
Illiterate – basic	49 (67)	70 (48)	2.2 (1.2–3.9)	1.3 (0.3–5.5)
Secondary – university	24 (33)	76 (52)		
Mother education				
Illiterate – basic	71 (97)	129 (88)	4.7 (1.1–20.8)	8.2 (0.4–157.7)
Secondary – university	2 (3)	17 (12)		
Nutritional status^a^				
Malnutrition	9 (19)	3 (5)	4.8 (1.2–18.7)	24.9 (1.9–329.6)^b^
Normal	39 (81)	62 (95)		
Vaccination status				
Unvaccinated	63 (86)	35 (24)	19.9 (9.3–43.1)	17.2 (2.9–100.7)^b^
Vaccinated	10 (14)	111 (76)		
Contact with measles case				
Contact	68 (93)	39 (27)	37.3 (14.0–99.2)	27.3 (1.3–551.7)^b^
Not contact	5 (7)	107 (73)		
Presence of measles case in the same area				
Yes	62 (85)	35 (24)	17.9 (8.5–37.7)	1.1 (0.1–19.2)
No	11 (15)	111 (76)		
House ventilation				
Bad	35 (48)	21 (14)	5.5 (2.9–10.5)	1.7 (0.4–7.2)
Good	38 (52)	125 (86)		
Distance between home and health facility				
≥ 3.3 km	21 (29)	57 (39)	0.6 (0.3–1.2)	3.8 (0.6–23.1)
< 3.3 km	52 (71)	89 (61)		
Crowding (Person per room)				
≥ 2.5	43 (59)	65 (45)	1.8 (1.0–3.2)	0.7 (0.2–2.5)
< 2.5	30 (41)	81 (55)		
Family size				
≥ 7	61 (84)	133 (91)	0.5 (0.2–1.2)	3 (0.4–23)
< 7	12 (16)	13 (9)		

^a^Number of children aged 6–60 months are 113 (48 cases and 65 controls)

^b^*P* value < 0.05

In multivariate analysis, malnourished children aged 6–60 months (aOR = 24.9, 95% CI:1.9–329.6), unvaccinated children (aOR = 17.2, 95% CI:2.9–100.7) and contacted patient with measles (aOR = 27.3, 95% CI:1.3–551.7) remained significantly associated risk factors with being a measles case.

## Discussion

Measles outbreaks exacerbated in Yemen as a result of ongoing war that lead to the collapse of the health system in all levels.

The result of this investigation revealed the highest peak of cases in week 21, this might be due to the aggregation of cases as a result of our investigation that was performed around this week. This indicated that the outbreak is still continuing in the affected area. Our finding shows that males were more affected than females. Our finding is consistent with previous investigations in Al Jawf and Sa’adah governorates [13, 18], and inconsistent with others in Amran governorate [15] and Ethiopia [19]. This is may be due to the fact that males are more mobile or socially active than females. Age group < 5 years was more affected; as a result of the war that led to collapse in the health system including lowering immunization coverage and the accumulation of the susceptible population in the age group < 5 years. This result is similar to studies in Al Jawf, Sa’adah, Amran governorates [13, 14, 16] and two in Ethiopia and Nepal [19, 25]. The higher AR is most likely due to the high susceptibility of population. Moreover, the overall AR in Habban was more than Ataq districts due to the un-vaccination status which was higher in Habban than Ataq districts. Furthermore, the immunization coverage in Habban (61 and 27%) is lower than Ataq district (93 and 66%) in 2017 and 2018, respectively [26]. Similar findings were found in several studies in Qufl Shamr district, Hajjah governorate and in Ethiopia [13, 27].

The result of the case control study shows the factors that related to spreading of measles infections among the population. Our investigation indicated that there isn’t a significant association between the children under-5 years and measles infection. This result is consistent with two studies in Ethiopia [28, 29]. Similarly, the children of illiterate or low educated fathers were found to be not significantly associated factor for measles infection. Our result agrees with study in Indonesia [30] and disagrees with study in Iran [22]. Moreover, the children of illiterate or low educated mothers aren’t significantly associated with measles infection. This finding agrees with study in Indonesia and Ethiopia [30, 31] and disagrees with study in Ethiopia [32].

Our results revealed that there is a strong association between malnutrition and measles infection. Malnourished children are at risk of measles infection 24 times than normal children. Our finding is consistent with studies in Pakistan and Ethiopia [21, 28] and inconsistent with other study in Ethiopia [32].

Regardless of vaccinated dose number, the results also reflect a strong significant association was observed between vaccination status and measles outbreak, therefore measles vaccine reduced the probability of getting measles infection 17 times during outbreak. This clearly suggests that measles infection is vaccine preventable. Our result agrees with two studies in Pakistan [21, 33], and others studies; one in Iran [22] and in Indonesia [30] and three in Ethiopia [29, 32, 34].

Additionally, there was found a strong association between the contact with measles cases. The individuals who contact with measles cases have a chance to get measles infection 27 times than those not in contact with the cases. This study result suggests the fact that measles virus has a secondary attack rate among susceptible individuals and the disease transmission by respiratory droplets in close case contacts. The result of this study is similar to previous studies in Pakistan, Southeast Iran, Indonesia and Zimbabwe [21, 22, 30, 35] and five studies in Ethiopia [28, 29, 31, 32, 34].

The current investigation indicates that there isn’t significant association between distance of health facility and measles infection. This may be because cases and controls were selected from the same place of residence. The result of this study is inconsistent with a previous two studies in Pakistan [21, 33]. We also found that there isn’t significant association between crowding and the chances of getting measles infection. This result agrees with studies in Ecuador, Ethiopia and Zimbabwe [20, 34, 35].

The study is a retrospective design in which recall bias might be raised. Use suspected instead of confirmed case definition to identify the cases in the field. Results of our study might not generalize to all governorates of Yemen because it was done in only one governorate.

In conclusion, measles outbreak in Ataq and Habban districts was confirmed. Contact with measles cases was the predominant contributing factor of measles outbreak in Shabwah governorate. As well malnutrition and un-vaccination were potential factors that contributed to the occurrence of this outbreak.

An urgent vaccination campaign with health education interventions are highly recommended. Health authorities and the World Health Organization should focus to reactivate the outreach immunization services and strengthen surveillance and response system at district and governorate levels.

## Data Availability

All relevant data are presented in this paper; and more information can be provided upon reasonable request from the correspondence author.

## Abbreviations

AR: Attack rate
aOR: Adjusted Odds Ratio
cOR: Crude Odds Ratio
CI: Confidence Interval
EMR: East Mediterranean Region
OR: Odds Ratio
